# Implementation of a Digitally Enabled Care Pathway (Part 2): Qualitative Analysis of Experiences of Health Care Professionals

**DOI:** 10.2196/13143

**Published:** 2019-07-15

**Authors:** Alistair Connell, Georgia Black, Hugh Montgomery, Peter Martin, Claire Nightingale, Dominic King, Alan Karthikesalingam, Cían Hughes, Trevor Back, Kareem Ayoub, Mustafa Suleyman, Gareth Jones, Jennifer Cross, Sarah Stanley, Mary Emerson, Charles Merrick, Geraint Rees, Christopher Laing, Rosalind Raine

**Affiliations:** 1 Centre for Human Health and Performance University College London London United Kingdom; 2 DeepMind Health London United Kingdom; 3 Department of Applied Health Research University College London London United Kingdom; 4 Population Health Research Institute St. George's, University of London London United Kingdom; 5 Royal Free London NHS Foundation Trust London United Kingdom; 6 Faculty of Life Sciences University College London London United Kingdom

**Keywords:** nephrology, acute kidney injury

## Abstract

**Background:**

One reason for the introduction of digital technologies into health care has been to try to improve safety and patient outcomes by providing real-time access to patient data and enhancing communication among health care professionals. However, the adoption of such technologies into clinical pathways has been less examined, and the impacts on users and the broader health system are poorly understood. We sought to address this by studying the impacts of introducing a digitally enabled care pathway for patients with acute kidney injury (AKI) at a tertiary referral hospital in the United Kingdom. A dedicated clinical response team—comprising existing nephrology and patient-at-risk and resuscitation teams—received AKI alerts in real time via Streams, a mobile app. Here, we present a qualitative evaluation of the experiences of users and other health care professionals whose work was affected by the implementation of the care pathway.

**Objective:**

The aim of this study was to qualitatively evaluate the impact of mobile results viewing and automated alerting as part of a digitally enabled care pathway on the working practices of users and their interprofessional relationships.

**Methods:**

A total of 19 semistructured interviews were conducted with members of the AKI response team and clinicians with whom they interacted across the hospital. Interviews were analyzed using inductive and deductive thematic analysis.

**Results:**

The digitally enabled care pathway improved access to patient information and expedited early specialist care. Opportunities were identified for more constructive planning of end-of-life care due to the earlier detection and alerting of deterioration. However, the shift toward early detection also highlighted resource constraints and some clinical uncertainty about the value of intervening at this stage. The real-time availability of information altered communication flows within and between clinical teams and across professional groups.

**Conclusions:**

Digital technologies allow early detection of adverse events and of patients at risk of deterioration, with the potential to improve outcomes. They may also increase the efficiency of health care professionals’ working practices. However, when planning and implementing digital information innovations in health care, the following factors should also be considered: the provision of clinical training to effectively manage early detection, resources to cope with additional workload, support to manage perceived information overload, and the optimization of algorithms to minimize unnecessary alerts.

## Introduction

### Background

In many health systems, the ageing demographic of hospital patients is accompanied by worsening health and a greater need for diverse investigations and treatments. As a result, care pathways are increasingly complex and ever more reliant on access to relevant data and on communication between individuals and multidisciplinary teams [1]. In this context, although adverse events—such as acute kidney injury (AKI), cardiac arrest, or clinical decline which necessitates high-dependency care—are commonly a consequence of the natural history of the underlying disease, they may also occur because of delays in treatment pathways [2]. Evidence suggests that patient outcomes improve where such clinical decline is detected and acted upon early, and substantial effort has been made worldwide in this regard, for instance, through the use of *track and trigger* scoring systems to detect decline [3] or the provision of emergency response teams [4,5]. However, such changes have not been matched by other key components of care delivery: the manner in which health care teams communicate and the manner in which data are accessed and presented. Globally, the most widely used hospital communication system continues to be the pager [6], whereas data are accessed from paper records and a range of disparate and disconnected electronic data repositories. Care might be improved if interpersonal communication were enhanced and if it were possible to readily access data in a form that allowed rapid assessment of the patient’s status.

The introduction of digital technologies offers one potential solution, allowing ready detection of the deteriorating patient, communication among health care workers, and immediate access to patient data in a user-friendly and appropriate format, thus improving outcomes. The embedding of digital technologies into health care is now a priority in the United Kingdom [7] and internationally [8]. However, the adoption of such technologies into health care has been little studied, and the impacts on users and the broader health system are poorly understood. We sought to address these issues by studying the impacts of introducing a digitally enabled care pathway for patients with AKI.

AKI—a sudden reduction in kidney function diagnosed by changes in serum creatinine [9]—is common, appears across multiple care pathways, and is associated with significantly increased mortality, morbidity, and cost of health care [10-15]. However, substantial deficits exist in all key processes of AKI care including early recognition and therapy, appropriate escalation to specialist or critical care services, and follow-up [16]. In an effort to expedite and standardize diagnosis, the National Health Service (NHS) mandated the use of a new diagnostic algorithm in all English hospitals in 2014 [17] and provided guidance as to how the algorithm could be implemented [18]. However, simple alerting to the presence of AKI does not seem to improve outcome [19]. We therefore designed a new care pathway that encompassed AKI detection, mobile alerting of a dedicated response team comprising multiple specialists, and the provision of protocolized care [20]. Evaluation of the implementation of the digitally enabled care pathway with regard to impacts on processes of care, clinical outcome, and the cost of care delivery are described elsewhere [21,22].

### Objectives

We present a qualitative evaluation of the experiences of users and other health care professionals whose work was affected by the implementation of the new care pathway. We sought to characterize the impacts on staff of such automated alerting, mobile results viewing, and ready communication, with particular focus on their working practices and interprofessional relationships.

## Methods

### Setting

The digitally enabled pathway was designed and implemented at the Royal Free Hospital (RFH)—a large, acute, tertiary referral hospital providing a range of acute services (including a 34-bed intensive treatment unit and an inpatient nephrology service) in central London, United Kingdom. The care pathway has been described in detail elsewhere [20] and is summarized in brief below.

#### The Preimplementation Care Pathway

Before making the changes to the care pathway described here, pathology results were viewed by ordering clinicians in batch at the end of the working day using desktop computers. A message linking to local Web-based clinical guidelines was appended to any creatinine result suggestive of AKI in the electronic patient record (EPR), and such results were also communicated to the clinical area by biochemistry staff by telephone. In its early stages, AKI was typically managed independently by general acute care and various specialty teams; specialist input was requested through hospital pagers and telephone communication at the discretion of referring clinical teams.

#### The Digitally Enabled Care Pathway

Streams (DeepMind Technologies Ltd) is a mobile app deployed on iPhone operating system–enabled smartphones. It processes relevant routinely collected clinical and demographic data through secure integration with hospitals’ existing information systems; owing to the need for real-time event-driven data, Health Level Seven (version 2, Health Level Seven International) feeds were used for integration with the laboratory information management system and electronic medical record. It was first registered with the Medicines and Healthcare Products Regulatory Agency as a Class I, nonmeasuring, nonsterile medical device under the European Union Medical Device Directive (1993) on August 30, 2016. Future revisions of the device may be classified at a higher level under the new medical device regulation.

Streams analyzes serum creatinine results immediately and continuously, alerting clinicians in real time to all potential AKI cases as defined by the NHS England AKI algorithm. The app also provides clinicians with data relevant to AKI management, including a graphical trend view of serum creatinine, specific flags for the presence of life-threatening AKI complications (such as hyperkalemia), details of any previous AKI episodes, demographic information, and past medical history from coded Hospital Episode Statistics data. Videos demonstrating Streams functionality can be found on the DeepMind Health support website [23].

AKI alerts are sent in real time to a specialist clinical response team (henceforth, the *AKI response team*), comprising the RFH’s existing *patient-at-risk and resuscitation team* (PARRT) and nephrology team. The PARRT (Clinical Nurse Specialists who review at-risk or deteriorating inpatients) receive alerts on all patients with AKI stages 2 and 3 and are on site 24 hours a day. The nephrology team comprises a renal consultant and specialty registrar, both of whom receive all AKI notifications. The registrar is on site 24 hours a day and is typically the first responder. The consultant can triage alerts through secure remote access if off site, providing clinical supervision and subsequent patient review where needed. Through Streams, the AKI response team triages alerts, communicates with other team members, and documents the outcome of clinical reviews. Relevant contacts and clinical guidance are available on Streams phones.

The AKI response team prioritizes patients for review according to the information available in Streams. Patients with life-threatening complications or deemed at high risk are reviewed immediately, whereas a case review within 2 hours is suggested for all other alerts. Upon review, a care protocol (based on existing best practice guidelines [24,25]) is annotated and entered into the patient’s paper notes alongside an advisory sticker for key nursing actions (Multimedia Appendix 1).

**Figure 1:**
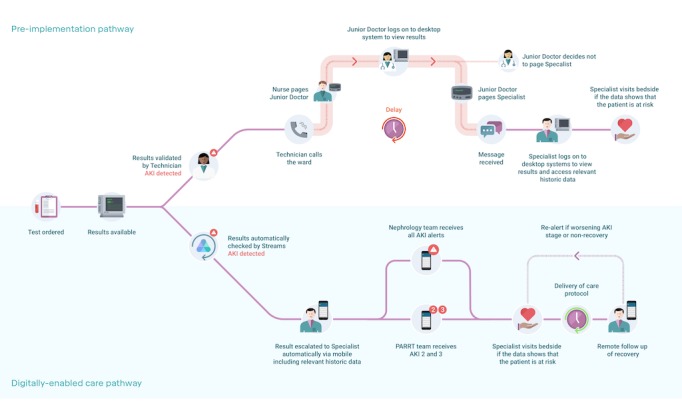
Pre- and postintervention care pathways. AKI: acute kidney injury.

Although the nephrology team occasionally takes over patient care, overall responsibility for care rests with patients’ primary teams. AKI recovery is monitored remotely in-app. Realerting for AKI that has not recovered is enabled 48 hours after the first alert. However, worsening of AKI stage or the development of a new complication (eg, hyperkalemia) at any time results in a further notification. Follow-up reviews are undertaken by the AKI response team according to clinical judgement. A diagram outlining the pre- and postintervention care pathways is provided in Figure 1. Although clinical guidelines and specialist response teams existed before the new care pathway, the goals of implementation of the care pathway were to improve the reliability and speed at which AKI recognition and appropriate specialist review occurred.

### Implementation

Before deployment, Streams users attended training events and accessed a video users’ guide to both the Streams app and the clinical pathway. Feedback from the AKI response team was gathered during a 16-week pathway optimization period (January-May 2017), during which key adjustments to the Streams app and associated clinical pathway were made. The optimized care pathway has been deployed continuously at RFH since May 2017.

### Data Collection

We undertook an exploratory case study approach to understand the implementation of Streams within a single site. Data collected included in-depth semistructured interviews and brief nonparticipant observations. The interview guide (included in the Multimedia Appendix 1) was developed by the research team, which included physicians with extensive experience in clinical nephrology and intensive care medicine and experts in health services research. Interviews explored the impacts of the care pathway on staff members and the care delivered to patients, with a particular focus on working practices and interprofessional relationships. Face-to-face interviews began 1 month after the start of the pathway optimization period and were spaced throughout a 16-month period of implementation and evaluation (February 2017 to May 2018). Purposive sampling was employed following a key informant strategy [26] that identified individuals with important roles in the environment under study who had expert knowledge to share impartially. A sample size of 20 is typical for a case study such as this, in line with both international consensus guidance and common practice in qualitative research [27-29]. Furthermore, the total number of users involved in providing the care pathway was small, which necessarily restricted the number of interviews that could be conducted. A list of potential respondents was drawn up to ensure representation from both groups in the AKI response team (ie, PARRT and nephrology teams) and clinicians from the wider hospital community affected by the care pathway, as well as a diverse range of clinical experience and level of comfort with mobile technologies. A total of 20 respondents were approached and 19 consented. In total, 8 PARRT nurses (5 *band 7* and 3 *band 8* or above) and 8 nephrologists (4 registrars and 4 consultants) were interviewed from the AKI response team. Of the respondents, 3 (2 consultant physicians and 1 medical registrar) from the wider hospital community were selected as a result of their frequent interactions with the AKI response team. Interviews were conducted by the lead researcher (AC). Each respondent was interviewed once. Respondents were informed that the interviewer was from a university and that the research was independent of the RFH. Field observations were undertaken during the first 4 weeks of the pathway optimization period. For these, the lead researcher observed user behavior in the emergency department and in inpatient wards during day and evening shifts using extensive note-taking to document users’ interaction with the Streams app and impacts on working practices and interprofessional relationships.

### Data Analysis

Data were analyzed using a combination of inductive and deductive thematic analysis techniques [30]. First, quotes from each interview were arranged into a matrix (Multimedia Appendix 1) in which rows represented individual respondents and columns represented categories that aligned to the basic principles of the intervention pathway (for example, the triage of AKI alerts). The matrix was populated by 2 researchers (AC and GB) who independently analyzed the entire dataset. Researchers met regularly to critique and challenge each other’s allocations; these were in turn reviewed with the lead researcher (RR), a process that enabled us to compare different professional groups’ perspectives and identify discordant views. The group then synthesized new descriptive codes based on emergent themes in the matrix (for example, the impact of real-time information availability) and assigned the quotes to these. Discordant quotes that challenged these themes were routinely sought and discussed. Additional independent oversight was provided by the lead researcher who identified additional quotes of relevance and refined the final themes. We employed the principle of *keyness* in our analysis [31], drawing out novel issues that might be generalizable and relevant to the adoption of digital health products in clinical practice (for example, how mobile working tools impact established clinical workflows). Our results present representative quotes for each theme, the titles of which were iterated through the writing process. We therefore took both a descriptive and an interpretive approach to analysis, first understanding how the intervention was used and how it affected clinical practice, then considering the intervention in terms of cultural practices and overarching *meta-themes* about mobile app use.

### Ethical Approval

The digitally enabled care pathway constituted a new standard service at RFH. Plans for the evaluation of the digitally enabled care pathway were independently reviewed by the University College London Joint Research Office. They directed that this study fell under the remit of service evaluation (rather than research), as per guidance from the NHS Health Research Authority [32]. As such, the service evaluation was registered locally with the RFH audit lead and medical director, and no participant consent was required. An independent data monitoring committee (which included a patient member) reviewed all analyses before preparation for publication. A full list of committee members is provided in the Multimedia Appendix 1.

## Results

Although interviews focused on the deployment of a pathway for patients with AKI, they also sought to more broadly characterize the impacts of automated alerting, mobile results viewing, and ready communication on staff, with a particular focus on their working practices and interprofessional relationships. In this respect, 3 central themes emerged.

### Theme 1: The Impact of Real-Time Information Availability

This theme relates to respondents’ experiences of the benefits and drawbacks of real-time mobile access to patient data. The app provided automated alerts about patients’ kidney function and gave staff access to current and historical information, such as previous pathology and coded diagnoses. These functions were reported to save valuable time among participants from both teams:

Being able to look up the blood results for anyone in the hospital wherever you are is unparalleled. [...] it feels almost archaic these days, to go and see a patient and then go and sit down in front of a computer 15 minutes later. As a doctor, you have to integrate what you know about them at the time of seeing them. So if you could literally have this phone, look at the results, go and see them... Or even look at it while you are seeing them. [...] It must save at least - I don’t know if you could analyze it - but it must save at least a couple of hours in a day.

Respondent 3: Nephrology team

This in turn expedited rapid intervention for deteriorating patients, wherever they were in the hospital:

The speed at which it happened was impressive. [...] I happened to be in A&E [Emergency Department] and got the alert of someone with severe kidney injury. [...] The patient was admitted to [...] a specialist renal ward [...] within 2 or 3 hours, which I don’t think would have happened without the app. [...] I think it streamlines care and speeds up the time in which they get a specialist renal review.

Respondent 9: Nephrology team

I personally have noticed [...] patients who have flagged up on the app that the clinical management has been poor up to that point. When we get involved, or the renal team get involved, that management changes [...] It has definitely saved people’s lives.

Respondent 14: PARRT

Being able to access all the bloods for the patients in the hospital and to be able to be alerted to the sick ones and already know about them before we usually do... Sometimes you know about them before the crash bleep comes through. You turn up and you think, “That was actually the alert I was coming to see.”

Respondent 10: PARRT

Participants in both teams found alerts to be particularly valuable for patients whose lead consultant was not a physician:

The most value came from patients under [...] surgical patients, for whom the list of priorities for their clinicians are very different from what [physicians] look for when they are looking after a patient. For those [patients], getting a rapid alert about deranged renal function is very valuable.

Respondent 6: Nephrology team

The provision of results and real-time clinical alerts and team communication via mobile phones introduced workload for clinicians in a new modality. Overall, experienced clinicians were able to integrate AKI alerts into their existing duties, discriminating between high- and low-priority cases and using this information to adjust their current priorities:

I would intermittently [...] check it, like I would [...] check emails, [...] check it every hour or so, something like that. And within 5 minutes or so I could easily flick through the alerts and [...] identify which ones I needed to see. [...] I felt it was very easy to use, I think some people when they were trying to use it would try and respond immediately to every alert. I wouldn’t personally, I didn’t think that was the best way to do it. Intermittently checking it throughout the day, I managed to keep on top of things.

Respondent 9: Nephrology team

However, some more junior clinicians in both teams felt that the pathway created additional workload and suggested that clinical review might not be deliverable to all patients by the AKI response team as configured currently:

It does increase our work. Some days [...] we can have eight or nine referrals. But there is obviously a huge issue about workload for many people. But if we need to increase the size of our team because of this then that’s a good thing. And also it highlights [...] the acuity of our patients in our hospital. These patients are not straightforward.

Respondent 19: PARRT

Others pointed out that the added burden of the app was related to the volume of *false positive* alerts produced by the mandated NHS AKI algorithm:

...if the noise of the system could be reduced it would be a lot better. If [we were] able to get rid of all the nonsense alerts, that would be fantastic.

Respondent 1: Nephrology team

Some respondents from the nephrology team pointed out that although important information was now more readily available, this created additional anxiety because it was not clear who was primarily responsible for delivering timely care. Once again, the need to expand the responsive workforce was suggested as a way to mitigate the workload-associated stress:

...as Renal [Registrars], [...] you are always now, in the back of your head, thinking “I’ve got this other job to do.” And I think it does create... not anxiety that keeps you up at night... But it’s another anxiety when you already have enough anxiety! So I think even if it was available in the hands of more people, or we were a bit clearer that during times of people being unwell who are your own patients, you shouldn’t prioritise Streams people because they are under another team, then that’s fine. That’s one way of dealing with it.

Respondent 3: Nephrology team

In summary, although the digitally enabled care pathway was widely valued for increasing efficient access to patient information, thus facilitating better care, some respondents reported increased workload and anxiety.

### Theme 2: The Implications of Early Detection

The digitally enabled care pathway was designed to expedite identification of AKI so as to avoid deterioration and improve outcomes, meaning that staff who normally respond to critically unwell patients would now also attend patients at lower risk. There was a divergence of opinion among members of the AKI response team about the rationale for this approach. Respondents in both teams pointed out that the AKI algorithm identified deteriorating patients at an earlier stage than was possible through other means:

It’s a good thing from the point of view that I know there are patients that are potentially sick out there. [...] You could have an AKI and look relatively well initially. But [...] nobody would have known about those patients.

Respondent 8: PARRT

Others emphasized the benefits of early recognition for patient health and in terms of reducing the complexity of required interventions:

I think it does a good job. We pick up patients that would maybe sit for another day or so before we pick them up. It’s certainly beneficial. It is more work, but we might be saving ourselves work in a couple of days, we might have to do more stuff to catch up.

Respondent 15: PARRT

However, some respondents in the nephrology team did not see the point of being alerted to low-risk patients:

...you get a lot of AKI stage 1s. They build up. Looking through those and dismissing them each time is time consuming. The AKI stage 2 and 3 [alerts] are more helpful for me to look at, so I tend to just look at those and dismiss the stage 1s.

Respondent 18: Nephrology team

Some respondents in this team also pointed out that early identification could not necessarily be aligned to early intervention because of a lack of knowledge with respect to appropriate management:

The patients you definitely need to see are the patients that have acute renal failure with a creatinine of 300 or 400 [µmol/L] that and it’s going up - patients you’d normally want to see [...] The other patients [...] that have a rising creatinine, but the creatinine is not very high - it doesn’t mean that they don’t need to be seen necessarily. We are not trained as doctors to look after those sorts of patients.

Respondent 1: Nephrology team

Thus, the shift toward earlier detection highlights the need to consider the resources required to manage both early and late disease and the training needed to enable clinicians to effectively intervene at an earlier stage.

### Theme 3: Behavioral Effects of the Care Pathway

The final theme demonstrates how real-time data provision affected relationships between users of the digital intervention and the broader health system with which the users interacted, together with how these changes impacted upon beliefs, behaviors, and care delivery. These are described in 3 subcategories below.

First, the digital care pathway affected behaviors *within* clinical teams in a number of ways. An immediate benefit was the use of mobile phones for team communication, experienced by members of both teams:

I’ve found the [mobile phone] really useful because I’ve been able to message my team when I’m out seeing a patient, rather than finding a phone and to bleep them with and waiting for them to answer.

Respondent 5: PARRT

However, a disadvantage was also identified: junior members of the nephrology team do not currently use the Streams app. Unequal access to patient data occasionally reversed the usual direction of communication of information from junior doctor to consultant, which some consultants suggested impeded learning opportunities:

I think it’s important for [Junior Doctors] to understand what the decisions about their patients are. They have to be across the data. And that’s why I prefer getting information from them [...] We were in the position where I was telling the Juniors what the blood results were. It makes me uncomfortable and it makes them uncomfortable.

Respondent 6: Nephrology team

In addition, the visualization of each other’s triage decisions within the app (a feature specifically requested by users) revealed the hitherto unrecognized variations that exist in professional judgements. Respondents in both teams raised the fact that knowledge of others’ decisions sometimes confused rather than clarified the clinical decision-making processes of colleagues:

I was quite surprised about how other people triaged initially. I felt we’d be much more similar in our thinking, because when we talk about other things we do think similarly about other stuff. [...] I felt like - probably naively - that everyone would do what I did. And they didn’t at all.

Respondent 11: PARRT

Second, the digital pathway had an impact on relationships *between* clinical teams. Several members of the nephrology team were uncomfortable about providing a clinical opinion when not solicited by the clinicians primarily responsible for that patient’s care:

So you might [...] call the team and say “we suggest you give some fluid,” but I don’t think it’s ethical to prescribe it yourself. After all, they might say, “Listen, he has heart failure.” So you can’t intrude.

Respondent 2: Nephrology team

However, there was an indication among several members of the PARRT team that this concern might be limited to communication between different specialty doctors:

I think the doctors have found it more difficult because in medicine there is this real model of, “[...] I don’t see this patient unless I’m asked to see them”. There’s this formality. [...] Nurses don’t think like that, people are used to us showing up. So it’s been easier for us to think of every patient that we see as our patient, our problem, our sick patients. I think that’s been easier for this team to absorb and deal with.

Respondent 11: PARRT

This is relevant because the type of information available also changed the professional group with whom this team directly liaised:

[I will] always [speak to] the nursing staff, because you are on their ward. It’s only polite and also you will generally be recommending frequencies of obs; they need to know what’s going on. And someone from the medical team. I would say the app is making me speak to more senior doctors more. [...] I’d be more likely to seek out a consultant and say “by the way, this person has alerted” and show them the app.

Respondent 5: PARRT

Third, the care pathway had an impact on the relationship between clinicians and their patients. In particular, several PARRT team members described that alerts identified patients at an earlier stage of AKI than was the case through established clinical pathways (eg, monitoring of vital signs). This may have led to an unexpected and evolving role for some members of the team. Several respondents described how the care pathway enabled them to help patients make informed decisions surrounding end-of-life care. For example:

Why do we have to talk about end of life just as I’m about to die? [...] We could plan. Every single person we’ve been referred today has a terminal disease. [...] Trying to move the decision making back, in a more timely way. [...] We are getting an alert before they have even triggered [via vital signs], so we can probably have a sensible conversation with a patient with capacity.

Respondent 4: PARRT

## Discussion

### Principal Findings

Qualitative and quantitative results from our mixed-methods service evaluation suggest that the digitally enabled care pathway has positive impacts on patient care [21,22]. Here, we demonstrated the ability to intervene in the treatment of deteriorating patients more quickly and the opportunity for earlier, more constructive end-of-life planning. The ability to integrate mobile results viewing into existing clinical workflows also appears to increase efficiency through the immediate access to specific and contextual information. However, this comes at a price, particularly for some junior staff, in terms of anxiety associated with increasing numbers of *priority* patients and information overload, in part exacerbated by false positive alerts. We also highlight the hitherto unrecognized need to ensure that relevant clinical specialties include training in prevention if we are to optimize the value of digital innovations that promote early intervention. These findings suggest that the true cost-effectiveness of such innovations cannot be assessed until the balance between early intervention leading to better outcomes and increased workload is ascertained. Maximization of utility also requires finding the most appropriate balance between sensitivity and specificity for clinical alerts. This can be difficult to achieve: although it is recognized that the NHS England AKI algorithm produces false positives, some argue that this is a necessary trade-off for enhanced sensitivity [33].

### Strengths and Limitations

This evaluation has a number of strengths. First, it benefits from the diversity of our respondent sample, presenting multiple perspectives on the intervention based on cultural differences between different professionals and teams. Second, the robust analysis uncovered issues that are likely to be generalizable to the implementation of other digital technologies in health care. Finally, our analysis team includes researchers from medical, public health, and psychology backgrounds, creating trustworthiness by encouraging debate and multidisciplinary interpretation.

Our evaluation has a number of limitations. First, we initiated interviews during the *pathway optimization* period to enable us to gather insights that would improve the pathway itself. However, interviews at this early stage may have been more prone to include negative feedback before users were used to the changes described. The period in which interviews were conducted did not allow us to assess whether perceptions of the care pathway changed over time. Second, interviews were conducted in a single clinical setting. Although our methods allowed us to identify the *active ingredients* of a digital intervention in an acute setting with communication systems familiar to health care teams worldwide, the magnitude of the efficiency benefits reported may vary according to the digital maturity of the health care environment studied.

### Comparison With Prior Work

Few studies of AKI alerting systems have been previously described. Alavijeh et al [34] used a questionnaire to assess satisfaction with a new automated AKI warning system among physicians working in primary care. The questionnaire used was limited to questions about respondents’ knowledge of the existence of the alert system, perceptions of its utility, and impact on practice, making comparisons with our findings difficult. However, in common with our findings, many respondents found the alert system *valuable*. Kanagasundam et al [35] examined the effects of an interruptive AKI clinical decision support system (embedded within an EPR system) through a series of semistructured interviews with stakeholders. Themes revealed were similar to those encountered in the generic clinical decision support literature, namely, alert fatigue and user dissatisfaction with mandatory interactions. Although respondents in Kanagasundam’s evaluation believed that the alerts led to earlier patient assessment, some clinicians found them to be an *insult to their knowledge*. A major reason for dismissing alerts was users’ need to review a comprehensive dataset (including historical creatinine) at the point of alert. We overcame this limitation by including a curated summary of relevant clinical data in-app at the point of alert. Kanagasundam et al also reported that the impression that the alert system prioritized sensitivity over specificity limited perceived credibility for some users. Our evaluation also demonstrated the presence of both uncertainties and variations in professional judgement among specialists, as to what changes in serum creatinine were clinically significant. In addition, respondents in our evaluation were occasionally unsure as to whether clinical intervention was warranted, even for cases where the AKI alert was perceived to be genuine. Bevan’s [36] exploration of the impact of a clinical decision support system for AKI embedded within a single hospital’s EPR found that alerts were unpopular because of their interruption to the established workflows. We were able to avoid this problem through the separation of alerts from the hospital’s EPR so that mobile working allowed users to integrate alert reviews into their routine working practice. Our finding that mobile working tools are integrated into clinicians’ workflow is pertinent given that junior medical staff currently spend almost half their working day using desktop computers [37]. Finally, the survey by Oh et al [38] of provider acceptance of automated electronic alerts for AKI demonstrated that approval of alerts was positively correlated with the belief that such alerts improved patient care and negatively correlated with the belief that alerts did not provide any novel information. The overall odds of approval decreased over time. Thus, the success of deploying clinical alerts is dependent on clinicians’ perceptions of their relevance. This tallies with our finding of diverse opinions about the value of low risk alerts. A number of mixed-methods analyses of electronic alerting systems for AKI are still underway; results from the qualitative segments of the *Acute Kidney Outreach to Reduce Deterioration and Death* [39] and *Tackling AKI* [40] studies are awaited.

### Conclusions

Our results are relevant to the design and evaluation of care pathways that involve automated alerts, mobile working, or the early deployment of specialist care. Such innovations will increasingly emerge with the application of machine learning [41,42] to early diagnosis and disease prediction. Our findings suggest that alerting systems aiming to encourage early or preventive action will achieve buy-in from health care professionals if they believe in the clinical and cost-effectiveness of the intervention, feel equipped to respond, understand clearly what their responsibilities are, and feel empowered to act. Training in prioritization of information is needed to balance the planned benefits of real-time access to mobile data with the cognitive load this will produce; digitally enabled pathways should be designed so that the most appropriate clinician is able to access the right data at the right time. E-alerting or the early deployment of a specialist resource may also have an impact on other clinical teams affected by implementation; future evaluations should seek to further explore this. Finally, the inevitable introduction of digital technology to health care is more likely to improve both patient outcomes and working practices if aligned with a commitment to proactively identify and address concomitant, and sometimes unexpected, sequelae.

## Appendix

Multimedia Appendix 1 Care Protocol, nursing advisory sticker, interview guide, and Royal Free Hospital committee members.

Multimedia Appendix 2 Matrix of quotes from interviews. Rows represent individual respondents, and columns represent categories which align to the basic principles of the intervention pathway.

## Abbreviations

AKI: acute kidney injury
EPR: electronic patient record
NHS: National Health Service
NIHR: National Institute for Health Research
PARRT: patient-at-risk and resuscitation team
RFH: Royal Free Hospital
