# Supplementary material for: Implementation of a Digitally Enabled Care Pathway (Part 2): Qualitative Analysis of Experiences of Health Care Professionals
Source: J Med Internet Res. 2019 Jul 15;21(7):e13143. doi: 10.2196/13143 (PMC6693304; doi:10.2196/13143)
Supplement: Multimedia Appendix 1 [file jmir_v21i7e13143_app1.pdf]

Royal Free London NHS
NHS Foundation Trust

This patient has generated an AKI alert on the Streams application.
The AKI response team are here to support and advise.
Ongoing management remains the responsibility of the home team unless otherwise agreed.

affix patient sticker here

We recommend the following actions to STOP-AKI:

**Management of life threatening complications of AKI**	**Critically ill:** Call PARRT (2525) or ITU (1030)	☐
	**Hyperkalaemia or acidosis:** commence medical therapy as per guidelines	☐
	**Fluid overload:** Commence diuretics, nitrates/oxygen (if necessary), fluid restriction	☐
**Sepsis and hypoperfusion**	**Sepsis:** complete Sepsis 6 care bundle	☐
	**Has an infection causing AKI:** send cultures, commence or escalate antibiotics	☐
	**Hypovolaemic:** Start bolus fluid protocol. Give 500mls crystalloid and reassess, repeat as necessary. Escalate to senior review after 2 litres bolus therapy	☐
	Commence maintenance IV fluids	☐
**Toxicity**	**Drug cessation or adjustment required**	☐
**Obstruction**	**Obstruction is possible and patient needs same day diagnostic renal USS** Please call Matteo Rossi for bedside USS on 07443101848. If out of hours then discuss with radiology (1462). If obstruction present please contact urology registrar on 1487 or on x39536	☐
**Primary Renal Disease**	**Perform urine dipstick**	☐
	If urine dip clear: order 'AKI diagnostic set (basic)' on Cerner	☐
	If blood or protein present: order 'AKI diagnostic set (glomerular)' on Cerner	☐
**General advice**<br>If in doubt, contact the AKI registrar on 07950860822 (day) or 07950843257 (night)<br>For guidelines and education, visit londonaki.net or download the London AKI app:	- Take 4 hourly observations & ensure an escalation plan is in place<br>- Commence a fluid balance chart, measure weights daily and set a daily fluid balance target<br>- Daily bloods: use 'AKI follow up' order set on Cerner and follow up to renal recovery<br>- Avoid contrast if possible. Consider prophylaxis where contrast absolutely necessary<br>- If renal function does not return to baseline at discharge, contact AKI registrar for advice	
**Follow up**	We will only see if contacted by you or re-alerted in Streams due to worsening AKI	☐
	We will schedule a further review	☐
	We will take over care of patient	☐

TIME SEEN:______:______ DATE: ______/_____/_____ SIGNED:________________________________

Grade: Registrar ☐ Consultant ☐ NAME:________________________________

Figure 1: the care protocol

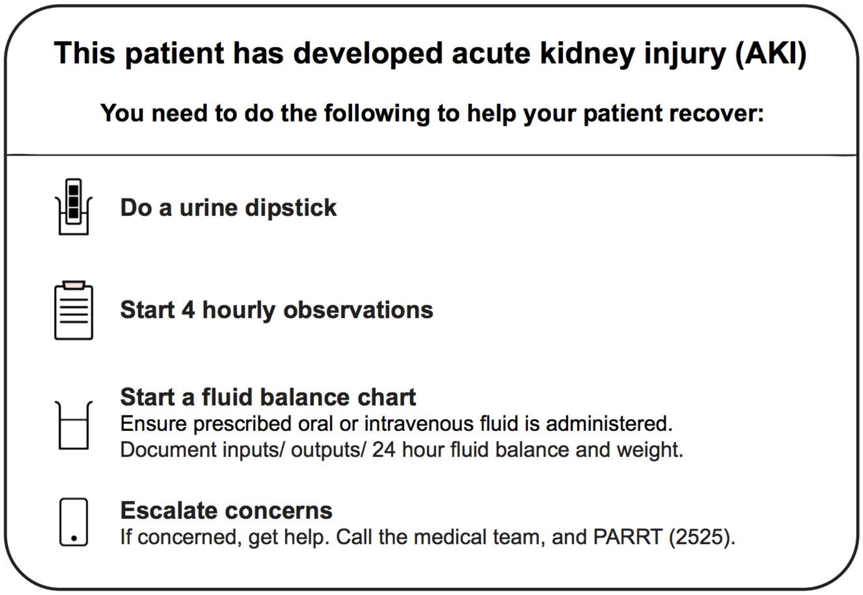

Figure 2: nursing advisory sticker

1. **Basic information**
    a. Are you a nephrologist or member of the PARRT team?
    b. What grade are you?
    c. How long have you worked at RFH?

2. **If part of the Streams-AKI Clinical Response Team**
    a. With respect to Streams...
        i. App: how easy or not was it to use the app? e.g. was it intuitive; when did you become comfortable with its use? What was bad?
        ii. Alerts: How did you feel about receiving alerts? What was good about the alerts? What was bad?
        iii. Triage: when/how did you prefer to triage? What were the barriers to this? What happened if you disagreed with someone else's triage decision?
        iv. Did you use the Streams phone for anything else?
    b. With respect to the proforma...
        i. Did you use it? If not, why not?
        ii. Any specific good & bad points to highlight?
    c. With respect to other team members...
        i. Was it useful being part of a response team?
        ii. Any frustrations with the way the team ran?
        iii. How did you contact other team members?
    d. What impact did the pathway have on you [good or bad]? Were there any unexpected effects?
    e. What impact did the pathway have from a patient point of view (good or bad)?
    f. What impact did the pathway have from the Trust point of view (good or bad)?
    g. Any other comments?

3. **If not part of the Streams-AKI Clinical Response Team**
    a. With respect to the Streams-AKI CRT:
        i. Good and bad- tell me about what changed in terms of
            1. your interactions with professionals who were part of the team?
            2. your patients management
        ii. What were the implications of this?
    b. What impact did the pathway have on you personally [good or bad]? Were there any unexpected effects?
    c. What impact did the pathway have from a patient point of view [good or bad]?
    d. What impact did the pathway have from the Trust point of view [good or bad]?
    e. Would you want the Clinical Response Team to keep using Streams? What would you change?
    f. Would you want to use Streams?

Figure 3: Interview guide

Chair

Prof. Kevin Moore
Professor of Hepatology, Royal Free Hospital

Patient representative

Michael Wise
Acute Kidney Injury National Programme Board

External members

Sir Nick Black
Professor of Health Services Research, London School of Hygiene and Tropical Medicine

Dr. Neil Ashman
Consultant Nephrologist and Deputy Managing Director, Barts Health

Royal Free Hospital Members

Dr. Jim Buckley
Consultant in Intensive Care Medicine

Dr. Nick Murch
Consultant in Acute Medicine

Dr. Jonathan Costello
Clinical Director, Emergency Medicine

Dr. Bimbi Fernando
Consultant in Transplant Surgery

Dr. Penny Smith
Consultant in Acute Medicine and Chief Medical Informatics Officer

Dr. Banwari Agarwal
Consultant in Intensive Care Medicine

Dr. Rupert Negus
Consultant in Acute Medicine and Gastroenterology

Figure 4: RFH Data Monitoring Committee
